# TESTING THE RELIABILITY AND VALIDITY OF THE RESIDENT SATISFACTION INDEX

**DOI:** 10.1093/geroni/igz038.2373

**Published:** 2019-11-08

**Authors:** Sarah D Holmes, Elizabeth Galik, Barbara Resnick

**Affiliations:** 1 University of Maryland Baltimore Doctoral Program in Gerontology, Baltimore, Maryland, United States; 2 University of Maryland School of Nursing, Baltimore, Maryland, United States

## Abstract

Understanding residents’ life satisfaction in assisted living (AL) is essential for creating supportive environments that are targeted toward the needs and desires of residents. Unfortunately, few measures have been developed and tested to evaluate residents’ life satisfaction in AL. The purpose of this study was to test the reliability and validity of the Resident Satisfaction Index (RSI) which was designed to measure residents’ life satisfaction in AL. Baseline data was used from a study testing the Dissemination and Implementation of Function Focused Care in AL. A total of 501 residents from 54 AL facilities were included in the sample. Based on Rasch analysis, there was evidence of internal consistency with an alpha coefficient of 0.95 and validity based on INFIT and OUTFIT statistics which ranged from 0.4 to 1.6. Item mapping showed the easiest item to endorse was item 10 which referred to perceptions of staff’s kindness. The hardest item to endorse was item 17 which asked about staff’s responsiveness to residents’ needs. Differential item functioning (DIF) analysis was done to examine differences in item responses by age, gender, and cognitive status. The measure was equally appropriate for those with and without mild to moderate cognitive impairment and between males and females, 5 out of 22 items were answered differently based on gender. 117 (23%) participants scored so high in satisfaction that they could not be differentiated. Findings support the reliability and validity of the measure although we recommend adding more difficult items to better differentiate between residents.

